# Anti-hypertensive Effect of Cereal Antioxidant Ferulic Acid and Its Mechanism of Action

**DOI:** 10.3389/fnut.2019.00121

**Published:** 2019-08-07

**Authors:** Md. Ashraful Alam

**Affiliations:** Department of Pharmaceutical Sciences, North South University, Dhaka, Bangladesh

**Keywords:** ferulic acid, hypertension, endothelium, reactive oxygen species, NADPH oxidase

## Abstract

Ferulic acid is a simple phenolic acid found mainly in cereals and grains, used as an antioxidant and food preservative. Recent evidence suggests that ferulic acid possess anti-inflammatory, anti-diabetic, anticancer, and cardioprotective properties. Several investigations also have shown that ferulic acid rich food might prevent hypertension. As a potent scavenger of free radicals (ROS, reactive oxygen species), ferulic acid attenuates oxidative stress, which is responsible for lowering elevated blood-pressure through improved endothelial function and increased bioavailability of the nitric oxide in the arterial vasculature. This review article describes the role of ferulic acid in the pathophysiology of vascular dysfunction and hypertension along with highlighted the merit of further scientific and clinical exploration.

## Introduction

Ferulic acid is a phenolic compound which is also known as a hydroxy-cinnamic acid derivative. Other compounds of this family include cinnamic acid, *p-*coumaric acid, caffeic acid, chrlorgenic acid, rosmarinic acid, and curcumin. *Ferula foetida* is the source of 3-methoxy-4-hydroxycinnamic acid which was isolated first by Austrian chemist Hlasiwetz Barth (1). Ferulic acid forms a structural component of lignocelluloses with dihydroferulic acid by cross linking lignin and polysaccharides which provides cell wall rigidity. Rice, wheat and oats, etc. are the major source of ferulic acid (1, 2). Other sources of ferulic acid are fruits and vegetables (1, 2). 3-(4-hydroxy-3-methoxyphenyl)-2-propenoic acid is commonly known as ferulic acid. Other names are 3-methoxy-4-hydroxycinnamic acid, caffeic acid 3-methyl ether, and coniferic acid (1). In most vegetables and fruits such as in coffee, cabbage, celery, and carrots, ferulic acid is conjugated with hydroxyl acids like quinic acid. In grains, ferulic acid may form an ester with sterols; gamma-oryzanol is one well-known example of this kind. Fifty to ninety percent of free ferulic acid is present in some vegetables such as burdock, water dropwort, and eggplant (3, 4). However, 0.1–0.5% free ferulic acid is present in cereals (4).

Ferulic acid is well-known for its antioxidant properties. Though ferulic acid was first isolated in 1866, its chemical synthesis procedure was discovered later in 1925 (1). Antioxidant properties of ferulic acid were first discovered by Japanese researchers in the extract from rice oil (5). Ferulic acid is used as a food additive antioxidant and food preservative in Japan (1). Chinese healers treat cardiovascular and cerebrovascular diseases using sodium ferulate, a salt of ferulic acid (6). The phenolic nucleus and carboxylic side chain present in the ferulic acid form a resonance stabilized phenoxy radical, which is responsible for the free radical-scavenging effect (1, 7).

In recent years, epidemiology investigations revealed the health benefits of ferulic acid-rich foods and drinks. Consumption of cereals, fruits, and vegetables are beneficial to prevent cancer, diabetes and cardiovascular diseases (8, 9). Moreover, ferulic acid has been shown to prevent type 2 diabetes, obesity, and Alzheimer's disease (7, 10). Several preclinical investigations also showed that ferulic acid improved glucose metabolism, insulin action, and lowered lipid parameters (11, 12). Investigations have also demonstrated that consumption of cereals and grains improved endothelial or vascular function and produced an anti-hypertensive effect (13). The presence of ferulic acid has been attributed to such biological effects, as an active component of cereals and grains. The purpose of this review is to highlight the health benefit of ferulic acid, mainly the effects and mechanisms of ferulic acid on hypertension and related disorder.

## Ferulic Acid Absorption and Bioavailability

**Figure 1:**
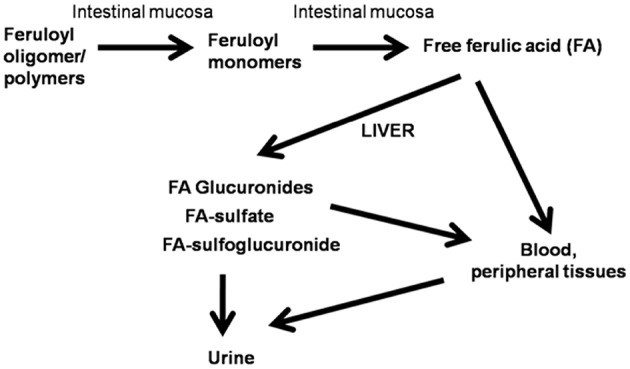
Schematic representation of absorption and metabolism of ferulic acid.

One third of the total dietary intake of polyphenolic compounds is consumed as simple phenolic acids. Considerable variation in the dietary intake of phenolic acids is seen in various geographic regions. Scalbert and Williamson estimated that subjects consuming fruits, vegetables, and phenolic acid-containing beverages in the daily diet, the daily intake of total polyphenols to be about 1,000 mg (14). Ferulic acid is absorbed relatively faster than another phenolic compound from the stomach (2). After absorption, ferulic acid is rapidly formed conjugation product in the liver with glucuronides, sulfate, and sulfoglucuronide (2) (Figure 1). Distribution of ferulic acid in the body after single administration is considerable. Orally administered ferulic acid was distributed ~4% in gastric mucosa, 10% in blood pool and liver, kidney, and 53% distributed in other tissues (2). However, free ferulic acid bioavailability is very low due to the rapid conjugation process in the liver (2). It is more bio-available than other dietary flavonoids and phenolics so far studied (15).

Ferulic acid is a relatively safe molecule having low toxicity. The LD_50_ value is 2,445 mg kg^−1^ body weight in male and 2,113 mg kg^−1^ body weight in female rat (16).

## Antioxidant Effect of Ferulic Acid

Ferulic acid showed strong antioxidant properties. The phenolic ring gives it strong resonance stability and can accept the electron more easily from free radical. It is thus a direct scavenger of free radicals. Moreover, hydrogen peroxide, superoxide, hydroxyl radical and nitrogen dioxide free radicals are scavenged very well by ferulic acid. Nitric oxide and ABTS^•+^ radical scavenging activity of ferulic acid are much better compared to caffeic acid (17). Hydroxyl radicals in a fenton reaction system were also scavenged almost completely by ferulic acid at a concentration of 250 mg/L (18).

Ferulic acid increases the antioxidant enzyme activity and function that are responsible for scavenging free radicals. It also inhibits free radicals producing enzymes in tissues. Previous studies showed that cardiac superoxide dismutase, glutathione peroxidase (GPx) and catalase (CAT) activities and mRNA expression were increased by ferulic acid and *p*-coumaric acid (19). Antioxidant enzyme activities were also increased in diabetic rats (11) and high fat diet fed mice (20). Another class of redox-sensitive inducible protein is heme oxygenase-1 (HO-1), that provides efficient cytoprotection against oxidative stress. Ferulic acid and its derivatives caffeic acid phenethyl ester (CAPE) and curcumin acts as a novel class of HO-1 inducer and protects cellular injury due to oxidative stress (21–23).

## Anti-inflammatory Effect of Ferulic Acid

Inflammation and immune response are considered as the key regulatory factors for the development and progression of various chronic diseases, such as cancer, diabetes, rheumatoid arthritis, atherosclerosis, and cardiovascular diseases. Ferulic acid showed anti-inflammatory action in various experimental *in vitro* and *in vivo* models. The LPS-treated Raw 264.7 murine macrophage is widely used to study inflammation *in vitro*. Macrophages play a central role in inflammation and are responsible for the production of inflammatory mediators such as pro-inflammatory and inflammatory cytokines (24). Ferulic acid prevented the production of macrophage inflammatory protein-2 (MIP-2) from RAW264.7 in response to respiratory syncytial virus (RSV) infection (25). Ferulic acid and its derivatives isolated from corn also inhibited inducible nitric oxide synthase (iNOS) expression in lipopolysaccharide (LPS)-stimulated Raw 264.7 cells as well as inhibited nitric oxide (NO) production (26). Similar results were also reported previously showed that inflammatory mediators, e.g., prostaglandin E_2_ and tumor necrosis factor-alpha (TNF-α) production was decreased by ferulic acid and related ester derivatives (18) and inhibited iNOS expression and function in lipopolysaccharide stimulated cells (27, 28).

Moreover, NCX 2057, a nitric oxide (NO)-releasing derivative of ferulic acid, inhibited iNOS mRNA and protein expression (29). The *in*-*vivo* anti-inflammatory response of ferulic acid was also reported in nicotine treated rats. Nicotine-treated rats showed increased expression of cyclooxygenase-2 and NF-κB in lung and liver which were reduced by ferulic acid (20 mg/kg) (30).

## Effect of Ferulic Acid on Diabetes and Hypercholesterolemia

Ferulic acid showed useful anti-diabetic activities (10). Blood glucose and plasma lipids were lowered successfully in STZ treated rats by ferulic acid (28, 31). *In vitro* intestinal alpha-glucosidase and porcine pancreatic alpha-amylase inhibitory activity have been reported recently by ferulic acid (32). In diabetes, an inhibitor of the intestinal alpha-glucosidase enzyme could be a useful treatment. Protective effect of ferulic acid was also seen in STZ rats by improving the antioxidant enzyme function (11). A recent report suggests that ferulic acid may increase the secretion of insulin from the cultured beta cell (32) and adiponectin from adipocyte (33). Improvement of glucose metabolism may be also responsible for the improvement of the vascular function. Ferulic acid supplementation improved the blood pressure in diet induced obese rats (34) possibly by improving the blood cholesterol and glucose metabolism (20). Previous reports also showed that ferulic acid decreased the hepatic HMG-CoA reductase, cholesterol transferase (ACAT), and cholesterol esterifying enzymes in the liver and exerted plasma lipid-lowering activities *in vivo* (26).

Cholesterol, especially LDL oxidation may be a potential source of developing the vascular diseases in diabetes and obesity. Ferulic acid showed potential inhibitory effect on LDL oxidation *in vitro* (35). Anti-atherogenic effect of ferulic acid was also seen in apolipoprotein E-deficient (apo E^−/−^) comparison with clofibrate in mice fed with a western diet (36). Ferulic acid also decreased the levels of phospholipids, lipid peroxides, low density lipoprotein, and very low density lipoprotein-cholesterol in the serum of isoproterenol intoxicated rats (37). Moreover, ferulic acid showed a protective effect on diabetic nephropathy. Supplementation with ferulic acid significantly prevented the rise of TGF-β1 mRNA expression in Otsuka Long-Evans Tokushima Fatty (OLETF) diabetic rat's kidney; however, ferulic acid showed no significant effect on COX-2 or ICAM-1 mRNA expressions (38).

## Effect of Ferulic Acid on Blood Pressure in Animals

Ferulic acid was used as an antihypertensive drug in Chinese medicine. The first antihypertensive effect of ferulic acid was reported in 2002 (39). Single dose (50 mg/kg) ferulic acid administration reduced the blood pressure of spontaneously hypertensive rats within the first hour and returned to the baseline within 6 h (39). This dose was comparable with captopril, an ACE inhibitor (dose 50 mg/kg). A positive correlation was also found with the reduction of BP and plasma ferulic acid concentration (39). Long-term supplementation of ferulic acid also reduced high blood pressure in L-NAME, a nitric oxide synthase inhibitor, induced SHR rats (39). However, ferulic acid showed no discernable effect in normotensive control Wister Kyoto (WKY) rats. In another study, rice bran fraction, rich in ferulic acid, prevented the elevated blood pressure in stroke prone SHR rats (34). Moreover, rice bran fraction lowered the urinary 8-hydroxy-2′-deoxyguanosine (8-OHdG) in Stroke prone SHR rats (34).

## Effect of Ferulic Acid on Vascular Function

Nitric oxide which may be produced from vascular endothelium, plays the crucial role in maintaining the vascular tone and blood pressure (40, 41). It is evident the excess free radical (ROS) generation is responsible for the development of endothelial dysfunction and hypertension (42–44). Excess free radical generation in vascular endothelium may scavenge the nitric oxide by forming more damaging peroxynitrile and reduces the NO bioavailability (44). The previous study showed that ferulic acid restored endothelial function in spontaneously hypertensive rat (SHR) aorta probably by enhancing the bioavailability of nitric oxide (NO) (45). This study also provided evidence that ferulic acid may relax the aorta even after the removal of the intact endothelium, which means a direct effect of ferulic acid on vascular smooth muscle cells (45). The endothelial independent vascular response was also evaluated by another group of the researcher. Chen et al. showed the vascular response is mediated via direct inhibition of the common pathway of smooth muscle contraction after [Ca^2+^]i increase by sodium ferulate (46). However, the direct vesoralaxant effect of sodium ferulate could also be mediated through the inhibition of PKC dependent contraction of smooth muscle (46). The hallmark signs of vascular diseases are the proliferation of VSMC with thickening of the intima and narrowing of the vessel lumen (47, 48). The previous study also suggested that vasodilators producing nitric oxide could inhibit the proliferation of rat aortic smooth muscle cells (49). Ang-II, an endogenous vasoconstrictor agent stimulates the VSMC proliferation and migration. Treatment with ferulic acid showed that VSMC proliferation due to angiotensin II was prevented in a dose-dependent manner (50). Later on, it was found that ferulic acid may potentially inhibit the Angiotensin converting enzyme (ACE), an enzyme facilitates the conversion of Ang-I to ANG-II (34).

## The Molecular Target of Ferulic Acid to Treat Hypertension

**Figure 2:**
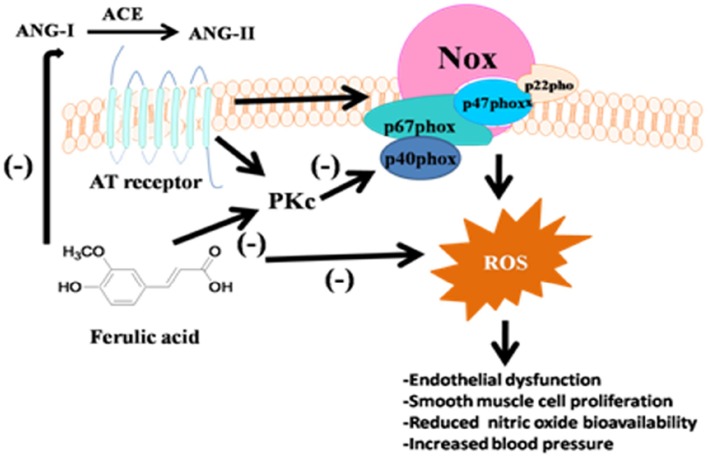
Schematic representation on the effect of ferulic acid on vascular response and oxidative stress.

How ferulic acid showed a beneficial effect on the cardiovascular system is not fully understood. Ferulic acid and its related derivatives have proven as a potent antioxidant and anti-inflammatory agent. The reactive oxygen species generation in vasculature have a direct correlation of developing hypertension in human and experimental animal (Figure 2) (43, 44). NADPH oxidase system present in the vasculature is the major source of free radicals (44). Angiotensin II has a profound effect on NADPH oxidase. Angiotensin II stimulates the ROS generation via NADPH oxidase in SHR rats (51, 52) and DOCA-salt rats (53) aorta. Suzuki et al. demonstrated that ferulic acid might inhibit the NADPH oxidase in SHR rat aorta (45). NADPH oxidase inhibitory activity may be evident as natural phenolic compound apocynine inhibits the NADPH oxidase. Interestingly, ferulic acid shares the core molecular structure of apocynine and thereby may acts as a non-selective NAD(P)H oxidase antagonist by reacting with sulfhydryl groups (54).

Another important target for ferulic acid might be the renin-angiotensin aldosterone system. Previous *in vitro* and *in vivo* studies showed that ferulic acid inhibited angiotensin-converting enzyme activity (34, 55). Single oral administration of ferulic acid (9.5 mg kg^−1^) in SHRsp rats showed that systolic blood pressure (measured by tail-cuff method) was lowered below the baseline, which was correlated to the simultaneous reduction of plasma angiotensin-converting enzyme activity from 20.6 to 16.9 mU ml^−1^ at 2 h (34).

Stress activated kinases are also involved in the proliferation and migration of VSMC. VSMC proliferation due to Ang-II stimulation is MAPK dependent. Several types of MAPK has been identified, ERK1/2, c-JUN kinase and P38 kinase system (56, 57). ERK1/2 and c-JUN kinase may directly be stimulated by ROS. Thus, it is evident that Ang-II mediated ROS generation may stimulate VSMC proliferation (58, 59). Ferulic acid inhibited the Ang-II mediated VSMC proliferation in a dose dependent manner (50). Ferulic acid also inhibited ERK1/2 and c-JUN kinase activity in VSMC after ANG-II exposure whereas the P38 MAP kinase was relatively unaltered (50). This study suggests that the inhibition of VSMC proliferation is mediated via ERK1/2 and c-JUN kinase mediated pathways.

Endothelium functions are regulated by the expression of specific genes in vascular endothelial cells. The physiologically-relevant concentration of polyphenol (ferulic acid, quercetin, and resveratrol) at a dose of 0.1 μM were studied in cultured human umbilical vein endothelial cells (HUVEC), followed by measurement of gene expression by microarray and quantitative RT–PCR. This study suggested that these selected polyphenols up regulated 233 genes and down regulated 363 genes (60).

Transcriptional regulator of the vascular cell was also modulated by ferulic acid supplementation. Nrf-2 is a key transcriptional factor that activates the antioxidant-reactive element (ARE). Nrf-2 and ARE also regulates the expression of antioxidant Phase II detoxifying enzymes. Ferulic acid supplementation may increase cardiac Nrf2 protein expression in rats (19) which may induce the antioxidant enzyme such as HO-1. Previously, we have discussed that ferulic acid may increase the antioxidant enzymes both in activity and mRNA level (19).

A recent investigation also reported that polyphenolics extract of whole wheat grains and ferulic acid protected doxorubicin-induced cardio-toxicity in rat cardiomyocytes (61). This study suggested that, ferulic acid prevented the increased iNOS expression, NADPH oxidase activation, Nrf-2/HO-1 impairment in doxorubicin-induced rat cardiomyocytes (61).

## Effect of Ferulic Acid on Cerebral Ischemia and Stroke

Cerebral ischemia is one of the deadly pathological conditions in which abrupt deprivation of oxygen and glucose is experienced by the brain tissues. As a result excessive production of free radicals, excess recruitment of inflammatory cells, and accumulation of intracellular calcium and initiation of apoptotic signals triggers ischemic damage to neuronal cells (62). Thus, antioxidants have been used for the development of neuroprotective drug in stroke therapy. Ferulic acid, as a small molecule, showed promising results (63). Previous study showed that rice bran extract supplemented with ferulic acid promoted functional recovery from ischemic injury in rat induced by middle cerebral artery occlusion (MCAO) (64). The reduction of cerebral infarct area and neurological deficit-score by ferulic acid treatment were at least partially attributed to the inhibition of superoxide radicals, and inhibition of inflammation by the reduction of ICAM-1 and NF-κB expression in transient MCAO rats (65). A recent study revealed that treatment with ferulic acid significantly attenuated memory impairment, and reduced hippocampal neuronal apoptosis and oxidative stress in a dose-dependent manner as well as inactivated TLR_4_ mediated inflammatory pathway (66). Protective effect of ferulic acid in neuronal apoptosis could be attributed to the activation of p38 MAPK mediated signal cascade which eventually inhibited the cytochrome c-mediated caspase-3-dependent apoptotic pathway (67). Nitric oxide (NO) acts as an important mediator in the brain and have both neuroprotective and neurotoxic effect in focal cerebral ischemia (68). Nitric oxide synthase proteins iNOS and nNOS expression levels have been seen increased during MCAO, and ferulic acid prevented the rise of these isoforms in rats (69).

## Future Direction

Ferulic acid showed very promising activities on blood pressure regulation and modulated many of the known physiological system and molecular mechanisms. It is still not clear, how ferulic acid increased the bioavailability of nitric oxide in the vasculature. No information is available to get direct evidence of increasing nitric oxide from eNOS system or increasing the eNOS both in protein and mRNA level. Another noticeable research area would be the cardiac structure and function. Few experimental results were found whether ferulic acid modulates left ventricular dysfunction and hypertrophy of heart both in human and animal study. Metabolic syndrome and vascular dysfunction are also observed in many of the obese and diabetes patients. Ferulic acid has the potential to improve glucose disposal and metabolic syndrome (11). Moreover, ferulic acid lowered the plasma lipids in experimental animal (11, 34). However, no information is still available about the beneficial effect of ferulic acid on cardiovascular function in metabolic syndrome.

## Conclusion

As discussed in this review, most of the beneficial effect of ferulic acid is due to its antioxidant and anti-inflammatory activities. However, the potential use of supplemental ferulic acid in the therapy of age-related human pathologies is still to be confirmed.

## Author Contributions

The author confirms being the sole contributor of this work and has approved it for publication.

### Conflict of Interest Statement

The author declares that the research was conducted in the absence of any commercial or financial relationships that could be construed as a potential conflict of interest.
